# DWSD: Dense waste segmentation dataset

**DOI:** 10.1016/j.dib.2025.111340

**Published:** 2025-01-31

**Authors:** Asfak Ali, Suvojit Acharjee, Md. Manarul Sk., Salman Z. Alharthi, Sheli Sinha Chaudhuri, Adnan Akhunzada

**Affiliations:** a Department of Electronics and Telecommunication Engineering, Jadavpur University, Kolkata 700032, India; b Department of Electronics and Communication Engineering, Narula Institute of Technology, Kolkata, India; c Department of Software Engineering, College of Computing, Umm AL-Qura University, Mecca 24381, Kingdom of Saudi Arabia; d College of Computing and Information Technology, University of Doha for Science and Technology, Doha 24449, Qatar

**Keywords:** Classification and segmentation, Computer vision, Smart cities, Waste management

## Abstract

Waste disposal is a global challenge, especially in densely populated areas. Efficient waste segregation is critical for separating recyclable from non-recyclable materials. While developed countries have established and refined effective waste segmentation and recycling systems, our country still uses manual segregation to identify and process recyclable items. This study presents a dataset intended to improve automatic waste segmentation systems. The dataset consists of 784 images that have been manually annotated for waste classification. These images were primarily taken in and around Jadavpur University, including streets, parks, and lawns. Annotations were created with the Labelme program and are available in color annotation formats. The dataset includes 14 waste categories: plastic containers, plastic bottles, thermocol, metal bottles, plastic cardboard, glass, thermocol plates, plastic, paper, plastic cups, paper cups, aluminum foil, cloth, and nylon. The dataset includes a total of 2350 object segments.

Specifications Table

**Table:** 

Subject	Computer Vision, Smart Cities, Waste management
Specific subject area	Classification and segmentation of the waste materials in the wild.
Type of data	Image.
Data collection	784 images are chaptered in the local area in West Bengal using a mobile camera. Most images were taken in and around Jadavpur University, Kolkata, Garia, Kolkata, West Bengal, and Berhampore, West Bengal including its streets, parks, lawns, and garbage area. The data was annotated using the Labelme [1] software in both Color annotation formats. There are 14 classes in the dataset, including 1. Plastic-container 2. Plastic-bottle 3. Thermocol, 4. Metal-bottle 5. Plastic-cardboard 6. Glass 7. thermocol-plate 8. Plastic 9. Paper 10. Plastic-cup 11. Paper-cup 12. Aluminium-foil 13. Cloth and 14. Lylon. The dataset contains a total of 2350 object segments. The dataset is named using the convention ``img +Sl No.png'' for the picture ground truth of color annotation.
Data source location	The Data are collected in various places in West Bengal: a. Jadavpur University, Kolkata, West Bengal b. Garia, Kolkata, West Bengal c. Berhampore, West Bengal
Data accessibility	Country: India Repository name: Mendeley Data Data identification number: 10.17632/gr99ny6b8p.1 Direct URL to data: https://data.mendeley.com/datasets/gr99ny6b8p
Related research article	None

## 1 Value of the Data

• The ‘DSWD’ is the first dataset for segmenting garbage from heavily populated areas, which is publicly accessible and it will serve as a benchmark dataset for the segregation of densely congested waste scenarios.
• This is the first waste segmentation dataset for the Indian garbage setting, to the best of our knowledge, which contains 2350 object instances, which will be helpful to train and test deep learning-based waste segmentation models.
• Except for TACO [4], the ‘DSWD’ has 14 waste classes more than most other available datasets, which are bio-degradable and non-biodegradable materials. This huge no of waste classes will help to test the robustness of the model in future research in waste classification and segmentation.
• The DWSD will be helpful for real-time waste segregation from the many types of mixed waste materials so that they can be recycled easily.
• In addition to creating the dataset, a benchmark is also developed for waste identification by utilizing various existing object segmentation techniques, such as UNet [14], DeepLabv3+ [13], FPNet [15], and PSPNet [16].

## 2 Background

Waste disposal has become a critical issue in all countries, especially the highly populated ones, and worldwide people are trying to segregate the waste efficiently so that materials that can be recycled are handled separately from the non-recyclable ones. Automated waste segmentation is the backbone of waste recycling in developed nations. However, in developing nations such as India, and Bangladesh, the waste separation process is mostly done manually, leading to serious health issues among the workers linked with solid waste management [14]. The researcher developed several datasets to attempt automatic waste segmentation systems. The Trash-ICRA19 underwater dataset for waste detection, which consists of 3 classes, was proposed by J. Hong et al. [2]. A Trashnet containing six different sorts of garbage was created by G. Thung et al. [3]. Additionally, J. Bobulski et al. [5] created the WaDaBa dataset, which classifies trash into 8 categories. Several more datasets, primarily for the classification of different types of waste, are also available on Kaggle, such as Waste Classification Data [6], Waste Classification Data v2 [7], waste pictures [8], and DeepSeaWaste [9], etc. Only the cigarette butt found in the wild is intended to be detected by a dedicated dataset called the cigarette butt dataset [12]. A limited number of datasets are available for trash segmentation. The TACO dataset has 28 classes, for garbage segments. A single-class indoor trash segmentation named MJU-Waste dataset was created by Tao Wang [11] et al. Marek Kraft et al. suggest using UAVVaste [10] to segregate garbage from drone images, which only have one class. One of the main problems with the existing dataset is that images from different European and American nations were used to compile it where waste is scarce. In contrast, Indian garbage scenes are highly congested and unorganized, making detecting them more challenging. This paper proposed a dataset for garbage segmentation, including data from both urban and rural West Bengal, India, making it more applicable to urban and rural garbage disposal. There are a total of 14 waste classification and segmentation classes in the proposed dataset. Both high and low-density waste situations were taken into account when creating the dataset. Table 1 displays a comparison of the proposed dataset with the current dataset. The existing datasets like UAVVaste and MJU-Waste v1.0 include only one waste segmentation class, our proposed dataset includes 14 waste classification and segmentation classes. Though TACO offers 24 classes, its images are less congested and do not feature the overlapping waste types that are typical in Indian scenes.

**Table 1:** Comparison of the existing datasets.

Name	No. categories	No. images	Annotation	Comment	Country
Trash-ICRA 19 [2]	3	5,700	Detection	Underwater images	Japan
Trashnet [3]	6	2,527	Classification	Clear background	United States
TACO [4]	24	1,500	Segmentation	Waste in the wild	-
WaDaBa [5]	8	4,000	Classification	Plastic dataset, clear background	-
Waste Classification data [6]	2	25,000	Classification	Scraped from Google search	-
Waste Classification Data v2 [7]	3	27,500	Classification	Scraped from Google search	-
waste_pictures [8]	34	24,000	Classification	Scraped from Google search	-
DeepSeaWaste [9]	5	3,055	Classification	Underwater images	-
UAVVaste [10]	1	772	Segmentation	Drone dataset	China
MJU-Waste v1.0 [11]	1	2475	Segmentation	Plain background, indoor	China
Cigarette butt dataset [12]	1	2,200	Detection	Waste in the wild, synthetic images	Austin, Texas
DWSD(Our)	14	784	Segmentation	Clear background, Waste in the wild(Rural and Urban area), synthetic images, Home-supplies	West Bengal, India

## 3 Data Description

The proposed DWSD contains 784 images with segmentation masks corresponding to each image. The high-resolution input images and crossposting ground truth segmentation mask with data description are shown in Table 2. Images are taken at different times of the day to incorporate various lighting conditions, ensuring a diverse range of illumination scenarios. All images are standardized to a resolution of 448 × 448 pixels. The dataset specifically targets densely congested waste materials, introducing a level of occlusion that is often not present in other datasets. This design choice enhances the dataset's relevance to real-world scenarios. Additionally, images are captured from both urban and rural areas surrounding water bodies and garbage sites, resulting in a wide variety of backgrounds. This variability contributes to the dataset's robustness, making it suitable for training models to recognize waste in diverse environments. The dataset contains 14 classes with 2350 object instances for segmentation. Although the dataset consists of high-quality images it has a very small number of images for training and testing any deep learning-based image segmentation. For that reason, the dataset is augmented to increase the number of images. First, each image is divided into 256 × 256 dimensions, and then augmentation techniques such as cropping, rotating, flipping horizontally and vertically, and blurring are applied.

**Table 2:** Example of the proposed dataset with segmentation mask.

Serial No.	Data Description	Input Image	Segmentation Mask
1	The image is taken in a rural area of West Bengal, this is an example of a densely congested waste image.		
2	The image is taken in an urban area of West Bengal, this is an example of a densely congested waste image.		
3	The image is taken in a rural area of West Bengal, this is an example of an uncongested waste image.		
4	The image is taken in an urban area of West Bengal, this is an example of an uncongested waste image.		

**Fig. 1:**
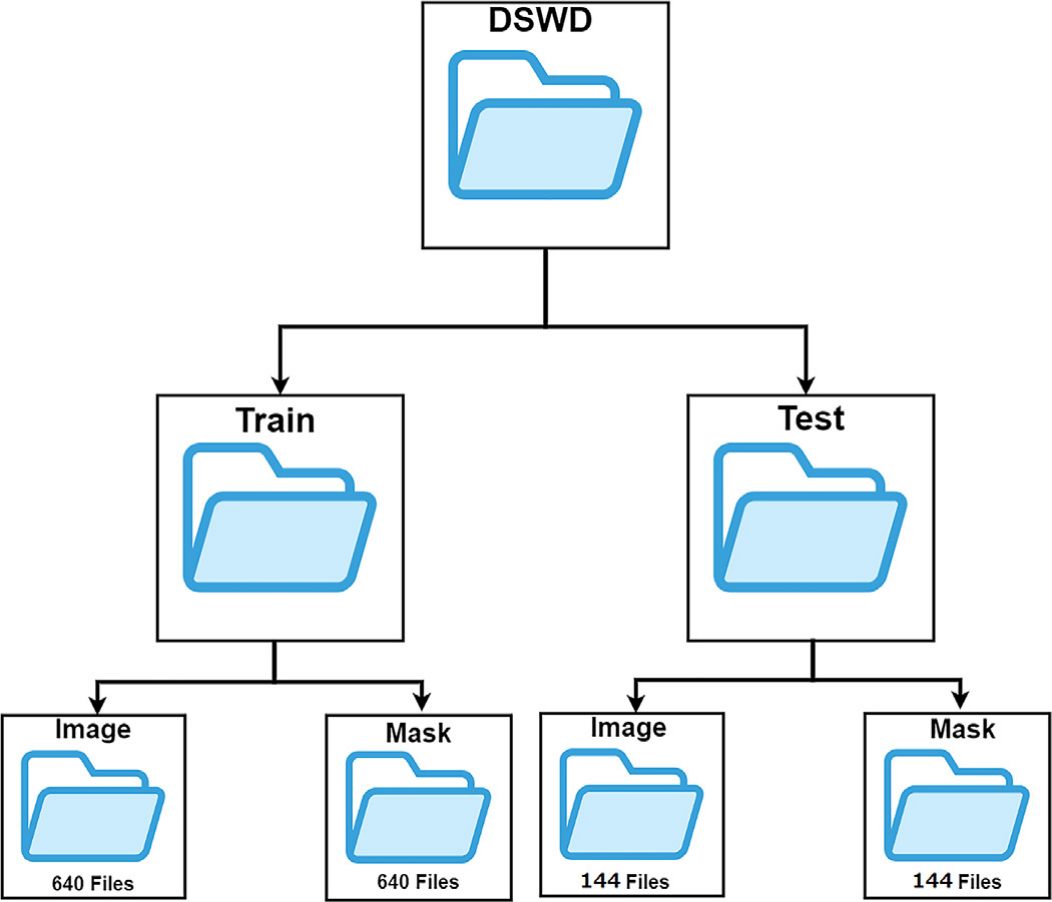
Data folder organization.

Then the dataset is divided into two parts, 640 images are for training and 144 for testing. The dataset folder consists of train and test folders. The train and test folder consists of the image and mask folder. The image folder consists of RGB images and the mask folder consists of ground truth mask images. The train image and train mask folder have 640 RGB images and corresponding ground truth masks respectively. On the other hand, the test image and mask folder have 144 RGB and ground truth images. The dataset folder structures of the ‘DSWS’ dataset are shown in Fig. 1. The mask consists of a pixel value of 0-14 representing each class of waste. In the dataset ‘0’ represents the background, ‘1’ denotes a plastic container, ‘2’ is for a plastic bottle, ‘3’ stands for thermocol, ‘4’ represents a metal bottle, ‘5’ denotes plastic cardboard, ‘6’ is for glass, ‘7’ stands for a thermocol plate, ‘8’ represents plastic, ‘9’ is for paper, ‘10’ denotes a plastic cup, ‘11’ stands for a paper cup, ‘12’ represents aluminum foil, ‘13’ is for cloth, and ‘14’ denotes nylon. Table 3 shows the class-wise pixel count in the proposed dataset.

**Table 3:** Class-wise pixel count in the dataset DWSD.

Pixel ID	Class Name	Pixel Count
0 and 15	Background	-
1	Plastic-container	22987
2	Plastic-bottle	8087
3	Thermocol	5075155
4	Metal-bottle	111194
5	Plastic-cardboard	3200244
6	Glass	111183
7	Thermocol-plate	494
8	Plastic	149592
9	Paper	65586
10	Plastic-cup	267732
11	Paper-cup	121085
12	Aluminium-foil	460122
13	Cloth	30658
14	Nylon	21895

## 4 Experimental Design, Materials and Methods

**Fig. 2:**
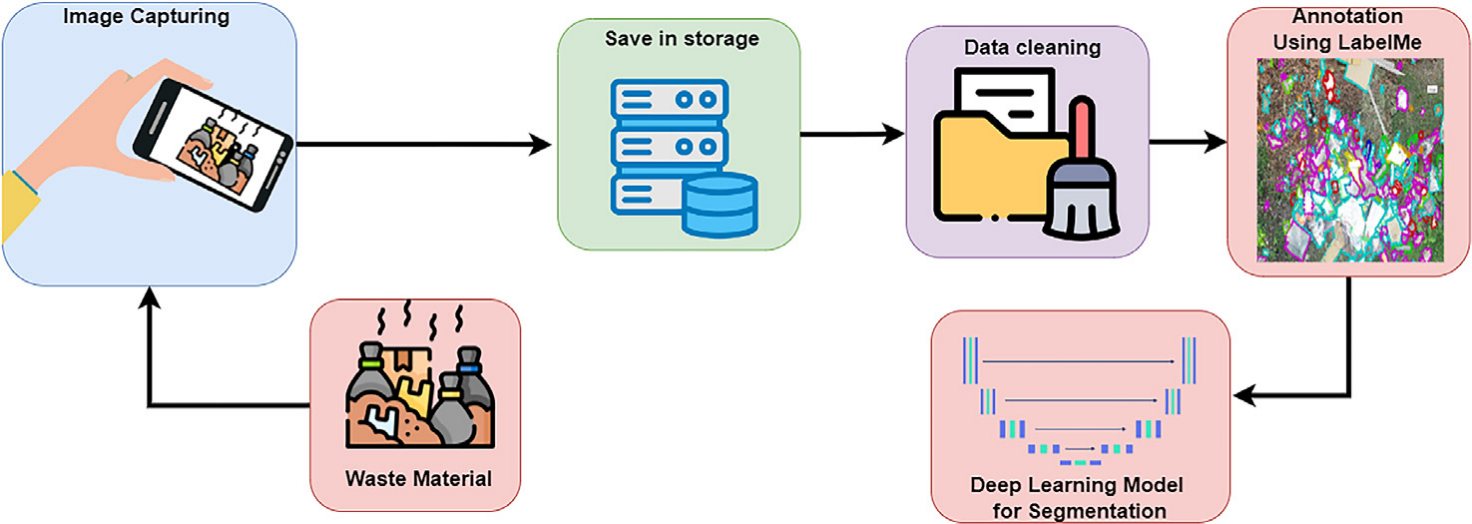
Flowchart of the dataset development process.

This section describes the dataset development process, which includes image capture, data cleaning, data annotation, and verification with standard deep learning-based image segmentation models such as DeepLabV3+, UNet, PSPNet, and FPNet. Fig. 2 shows a flowchart of the dataset development process.

### 4.1 Image capturing

The raw data for the 'DSWD' is captured using a mobile camera equipped with a 16MP Rear Camera Sony IMX519 image sensor and a 64MP Samsung GW1 imaging sensor. In total, 1000+ images were captured with 1920 × 1080 dimensions. The majority of the photographs were taken in and around Jadavpur University, Kolkata, Gariya, Kolkata, West Bengal, and Berhampore, West Bengal, including the streets, parks, lawns, and garbage dump. During image capture, different light conditions, angles, and waste scenarios are considered for data variation.

### 4.2 Data cleaning

Before the annotation process, the data is cleaned. Initially, 1000+ images were captured for the dataset creation, and high-quality images were chosen. Images with noise, poor quality, blurriness, overexposure, and low resolution are removed from the dataset, leaving 784 images for annotation. The goal of data cleaning is to ensure accurate data collection while also producing a refined dataset.

### 4.3 Data annotation

After data cleaning, manual annotation is performed using the LabelMe [1] software. The dataset consists of 14 classes, with objects segmented by selecting their regions within the images, ensuring high-quality segmented masks. An example of the annotated data is provided in Table 2.

The annotation process follows clearly defined class criteria, with specific guidelines to ensure consistent inclusion. In instances where an object could belong to multiple categories, detailed instructions are provided to guide annotators in selecting the most appropriate class. All annotators undergo comprehensive training on the dataset categories, annotation tools, and best practices. A well-structured manual is provided to explain how to label objects, manage occlusions, and resolve ambiguous cases. Precise delineation of object boundaries is required to prevent overlap with adjacent classes, ensuring clear segmentation. In cases where objects are occluded, annotators focus on the visible segments to capture as much information as possible, with annotations reflecting the true boundaries of the objects based on contextual clues. When objects overlap, only the visible portions are annotated to ensure accuracy. A consensus review process involving multiple annotators is implemented for difficult or ambiguous cases to maintain consistency and precision in the labeling. Regular feedback is provided to annotators to improve their performance, and the guidelines are continuously updated to address any emerging challenges or ambiguities.

A key challenge during annotation arises from the dense congestion of waste materials, where multiple objects overlap or occlude one another, making it difficult to distinguish individual waste types. Additionally, low-light conditions present issues in accurately identifying object boundaries, and complex backgrounds, such as those in garbage sites or near water bodies, further complicate segmentation.

A multi-step approach is employed to address these challenges. Annotators are instructed to segment even partially visible objects, especially in occluded regions, to capture as much object detail as possible. In low-light scenarios, image enhancement techniques are applied to improve visibility before annotation. Automated quality checks cross-verify the annotations against the original images to detect any inconsistencies. The annotations undergo review by two levels of annotators to reach consensus and improve accuracy. These measures mitigate the challenges posed by densely congested waste, occlusion, and poor lighting, ensuring a high degree of accuracy in the final annotations.

### 4.4 Model training and testing

To test the integrity of the proposed ‘DSWD’ dataset, four object segmentation models, such as DeepLabv3+, UNet, PSPNet, and FPNet, are trained and tested.

#### 4.4.1 DeepLabv3+

**Fig. 3:**
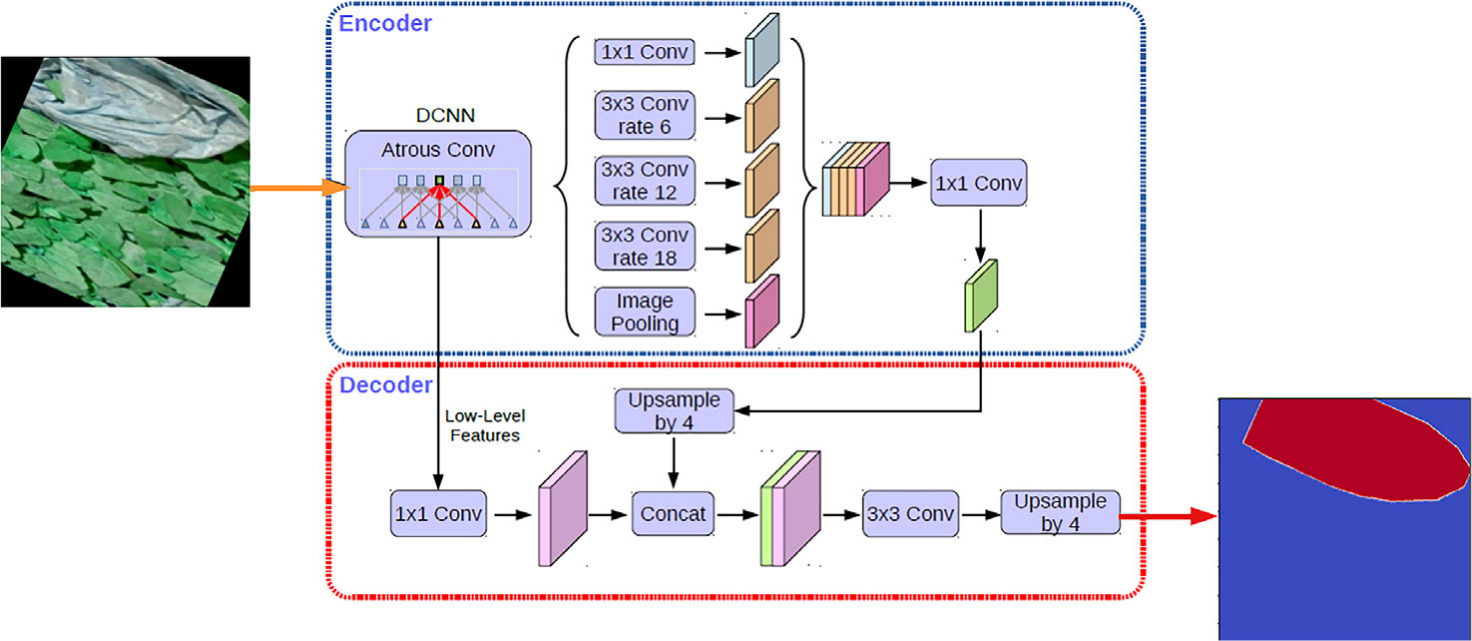
Architecture of the DeepLabV3+.

The DeepLabV3+ model for multi-class semantic segmentation is a fully convolutional architecture that performs well on semantic segmentation benchmarks. A simple yet efficient decoder module is added to DeepLabv3+, a semantic segmentation architecture, to improve segmentation results over DeepLabv3. The architecture of the DeepLabv3+ is shown in Fig. 3.

#### 4.4.2 UNet

Olag Ronneberger et al. designed the UNet architecture for biomedical image segmentation. The novel presented architecture was built around two key components: the encoder and the decoder. Covenant layers come first in the encoder, followed by pooling operations. It is used to extract image factors. The second part decoder uses transposed convolution to enable localisation.

#### 4.4.3 PSPNet

Pyramid Scene Parsing Network, or PSPNet, is a semantic segmentation approach that employs a pyramid parsing module to leverage global context data via context aggregation based on distinct regions. When local and global hints are combined, the final forecast becomes more accurate.

#### 4.4.4 FPNet

FPNet is designed for lightweight and low-power portable devices, consisting of a small number of parameters. FPNet is designed using a superblock based on dense residual blocks and a squeeze-and-excitation unit.

**Fig. 4:**
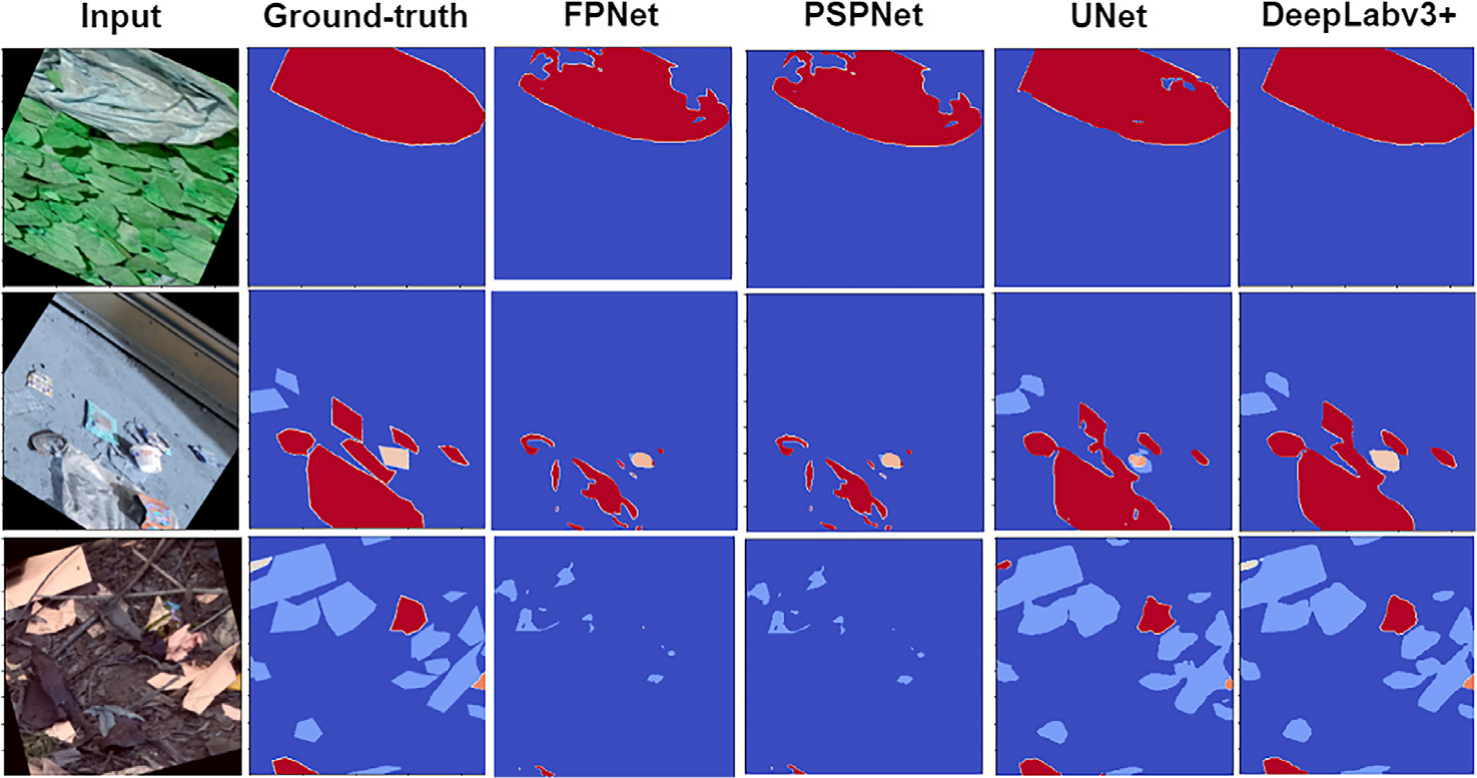
Qualitative result of four deep-learning-based segmentation models FPN, PSPNet, UNet and DeepLabV3+.

The models are trained and tested on a CoLab Notebook equipped with 51 GB of RAM and a 32 GB Nvidia Tesla P100 GPU. The models are trained over 150 epochs. With only 52 images, the augmentation technique was used to generate data for deep learning model training and testing. First, each image is divided into 256 × 256 dimensions, and then augmentation techniques like cropping, rotating, flipping horizontally and vertically, and blurring are used. These augmentations help reduce potential biases and prevent overfitting during training. The training folder contains 640 images, while the test folder contains 144. Table 4 compares the accuracy of training and testing, the DeepLabv3+ produces the best results in terms of testing accuracy. Fig. 4 shows the qualitative comparison of different models, where DeepLabv3+ shows better results.

**Table 4:** Quantitative experiment results.

Model Name	Training Accuracy	Testing Accuracy
DeepLabv3+	98.12%	89.39%
UNet	97.28%	88.99%
PSPNet	89.80%	83.80%
FPNet	87.50%	81.33%

## Limitations

The ‘DSWD’ dataset is the first waste segmentation dataset for dance-congested waste in the Indian subcontinent, and it is extremely useful for automatic waste segregation and classification. It has 14 classes and 2350 instances of waste objects. However, this paper only proposes 784 images for annotations because waste segmentation annotation is a time-consuming and labor-intensive task. The smaller amount of data may not be useful for deep-learning model training, so we cropped it and generated 784 images for training and testing the deep-learning model, after which we published the augmented data for future research.

Several promising avenues exist for expanding and enhancing the utility of this dataset. A primary focus will be on increasing the number of images to encompass a wider range of waste disposal environments across diverse geographical regions, both within India and internationally. This expansion will enrich the dataset's diversity and bolster its applicability to various real-world scenarios. Additionally, we intend to introduce more waste categories, addressing specific types of waste that are prevalent in different regions but currently underrepresented in the dataset. This enhancement will contribute to the accuracy and granularity of waste classification and segmentation tasks, facilitating more effective waste management strategies.

Furthermore, we plan to incorporate annotations for various environmental conditions, such as differing weather patterns, lighting conditions, and seasonal variations. By simulating the real-world challenges faced by waste management systems across various climates, these improvements will significantly enhance the dataset's relevance and utility for researchers and practitioners alike.

In conclusion, the dataset presented in this study offers significant potential for advancing automatic waste segmentation systems, a crucial step toward improving waste management practices. Given the global challenge of waste disposal, particularly in densely populated areas, effective waste segregation is essential for differentiating recyclable materials from non-recyclable ones. While developed nations have implemented efficient waste segmentation and recycling systems, manual segregation remains prevalent in many regions, including our country. This dataset, consisting of 784 meticulously annotated images featuring 14 waste categories, serves as a valuable resource for training machine learning models to automate waste classification processes.

The application of this dataset can lead to several impactful use cases in real-world waste management systems. For instance, municipalities can integrate automatic waste segmentation systems powered by models trained on this dataset into their waste collection and sorting processes. This integration can enhance efficiency by reducing the reliance on manual labor and increasing the accuracy of waste classification. Adopting automatic waste segmentation systems trained on this dataset could significantly impact both local and global waste management practices. Locally, such systems can streamline the waste sorting process, leading to higher recycling rates and a reduction in landfill contributions. On a global scale, widespread implementation of these technologies can contribute to better resource recovery and environmental sustainability, aligning with international goals for reducing waste and promoting circular economies. However, the transition to automated waste segmentation systems also presents challenges. Ensuring that models generalize well across different waste types and environmental conditions is essential for their success. Additionally, there may be initial resistance from stakeholders accustomed to traditional methods, as well as the need for adequate infrastructure to support the integration of new technologies.

## Ethics Statement

The authors have read and followed the ethical requirements for publication in Data in Brief and are confirming that the current work does not involve human subjects, animal experiments, or any data collected from social media platforms.

## Credit Author Statement

**Asfak Ali:** Conceptualization, Data curation, Formal analysis, Methodology, Writing – original draft; **Suvojit Acharjee**: Investigation, Writing & editing, Visualization; **Md. Manarul Sk.:** Data curation, Formal analysis, Conceptualization, Methodology; **Salman Z Alharthi:** Data curation, Formal analysis, Writing & editing, **Sheli Sinha Chaudhuri:** Resources, Project administration, Supervision, **Adnan Akhunzada:** Writing & editing, Resources.

## Data Availability

Mendeley DataDWSD: Dense Waste Segmentation Dataset (Original data).
